# Pepsinogen Serology and Gastritis OLGA Staging in Mucosal Atrophy Assessment: A Cross-Sectional Study Involving East China Endoscopy Population

**DOI:** 10.1155/2020/2324505

**Published:** 2020-04-13

**Authors:** Junjun Xia, Zhenzhen Liu, Kaiguang Zhang

**Affiliations:** Department of Gastroenterology, Anhui Provincial Hospital, An Affiliated Hospital of Anhui Medical University, 230000, China

## Abstract

The histological gastric stage (OLGA) plays an important role in evaluating gastric atrophy, a symptom which suggests a risk of gastric cancer (GC). However, gastroscopy is an invasive examination, which has limited application in populations undergoing physical examination. Serum pepsinogen (PG) and gastin-17 (G-17) levels are noninvasive indexes which are recommended when screening for GC. We aim to explore the correlation between PG/G-17 and OLGA stage, in order to provide reliable indexes for GC screening. The study included 453 asymptomatic individuals from East China undergoing physical examination, who then underwent endoscopy including collection of biopsy samples. Assays for serum PG, G-17, and *Helicobacter pylori* (Hp) were performed. Atrophy of gastric mucosa was graded according to OLGA for each individual. 453 participants, average age 52.46 ± 10.30 years, 253 male and 200 female, were included. In the asymptomatic physical examination population, serum PGI, PGII, and PGR levels decreased with increasing OLGA scores. PGI and PGR were inversely correlated with increasing OLGA stage in both Hp-positive and Hp-negative groups. The levels of serum PGI, PGII, and G-17 in the Hp-positive group were higher than those in the Hp-negative group; conversely, the PGR levels were lower. Furthermore, OLGA scores increased with age in the Hp-positive group. In conclusion, there is a significant correlation between OLGA stage and serum PG in populations from East China undergoing physical examination. Serum PG and G-17 combined with Hp test plays an important role in evaluating gastric atrophy.

## 1. Introduction

Gastric cancer (GC) is the fifth most common cancer in the world.

Approximately 1 million GC cases are newly diagnosed and almost 700,000 people die from this disease annually, which accounts for 10% of the world's cancer-related deaths [1]. The areas with the highest incidence of GC are Eastern Europe, East Asia, and parts of central and Southern America, while with the lowest in Southern Asia, North and East Africa, Australia and North America [2]. GC is the second most common cancer in China, after lung cancer [3]. Early gastric cancer has a better prognosis, with a 5-year survival rate of more than 90%, while the 5-year survival rate of advanced gastric cancer is less than 40%. To reduce the incidence of GC, early diagnosis and active intervention are essential.

Among all possible risk factors, *Helicobacter pylori* (Hp) infection is a major causal factor for GC. Under the influence of long-term Hp infection and other factors, gastric mucosa can undergo the processes of atrophy, intestinal metaplasia (IM), dysplasia, and finally GC [4].

The international atrophy research team proposed the assessment standard of inflammation and atrophy degree and scope of gastric mucosa (i.e., OLGA stage of chronic gastritis) in 2005, which links the histopathology of chronic gastritis with cancer risk. OLGA stage is able to rank the risk of GC by combining the degrees of atrophy in antral mucosa and corpus mucosa. Trials conducted in cross-sectional and cohort studies revealed that GC is associated with OLGA stages III and IV, which are defined as high-risk stages [5, 6]. However, endoscopy is an invasive examination, and OLGA stage, which needs extensive sampling, is influenced by pathologists, so it is not ideal in clinical practice, especially in healthy physical examination populations. Therefore, there is a need to screen for atrophy and high risk of gastric cancer using a noninvasive method in these populations.

It is universally accepted that serum pepsinogen (PG) and gastin-17 (G-17) levels reflect the functional and morphologic status of gastric mucosa. Serum PG levels contribute to the diagnosis of atrophic gastritis (AG) and can be applied to GC screening using the indicators PGI and PGR, levels of which associate with AG and with GC, respectively.

Therefore, the combination of serum PG and Hp infection has been suggested as a predictive marker for patients with GC [7]. Previous studies have stated the correlation between serum PG levels and OLGA stage [6, 8, 9], but few studies have been reported in asymptomatic populations undergoing physical examination in East China. It is generally accepted that Hp is a high-risk factor for GC, but reports on the effect of Hp on the relationship between serum PG levels and OLGA stage are rare.

This study is aimed at evaluating the correlation between serum PG and OLGA stage in evaluating gastric mucosal atrophy of the asymptomatic population, in order to define a simple and effective screening method to reduce mortality from GC.

## 2. Methods

### 2.1. Study Population

This cross-sectional descriptive study was conducted at the Anhui Provincial Hospital, an affiliated hospital of AHMU (Anhui Medical University). The study population consisted of 453 asymptomatic individuals from East China undergoing physical examination, who underwent endoscopy with biopsy sampling between 2015 and 2018. Serum PG/G-17 tests and Hp detection were performed simultaneously. The exclusion criteria were subjects with previous history of upper gastrointestinal surgery; patients using medication for stomach diseases, such as proton pump inhibitors (PPI), in the week before inspection; patients with serious lesions in important organs; patients with autoimmune diseases and patients with space-occupying lesions that might affect the analysis. What is worth mentioning is that all participants did not receive Hp eradication therapy before physical examination.

### 2.2. Endoscopy and Pathology

Gastroscopy examination and pathological biopsy sampling were performed by experienced physicians who were blind to the serological data of the subjects. Pathology samples were taken based on the Sydney system: two from the gastric body; one from the mucosal of the angularis incisura, and two from antral mucosa. All biopsies were fixed in 10% formalin, embedded in paraffin, and then sectioned and stained in a pathology laboratory. Each biopsy was scored by two pathologists according to the Sydney classification system [10] and was then assessed in combination with the atrophy of antrum and gastric mucosa.

### 2.3. Serum Pepsinogen Test and *H. Pylori* Status

Approximately 5 ml fasting venous blood was collected in the morning from each participant and stored at 4°C for 24 hours. After the samples were centrifuged at 3000 rpm for 10 min, the serum was aliquoted and frozen at -20°C, then shifted to -70°C before assay. Levels of serum PGI, PGII, and G-17 were measured by ELISA assay (Biohit Co., Helsinki, Finland), and PGR was calculated.

Each participant underwent a ^13^C-urea or ^14^C-urea breath test on the same day as endoscopy. A diagnosis of Hp infection was based on the results of the breath test combined with a pathological examination.

### 2.4. Statistical Analysis

When data showed a normal distribution, the results were represented by mean ± standard deviation (SD). Abnormally distributed data were expressed as median ± quartile. Statistical analysis was carried out using SPSS 22.0 software (SPSS Inc., Chicago, USA). The significant differences of age and serum PG levels among different groups were assessed by analysis of variance (ANOVA) and a Scheffe test. Hp infection differences were evaluated by Pearson's *χ*^2^ test.

A *p* value of less than 0.05 was regarded as significant.

## 3. Results

### 3.1. Participants' Characteristics

A total of 453 subjects were enrolled in the study, and the baseline characteristics of the participants are presented in Table 1. The age range of the subjects was 22 to 80 years. The mean age of the patients enrolled was 52.46 ± 10.30 years, 47.7% were male, and the mean body mass index (BMI) was 23.89 ± 3.29. The rate of Hp infection was 47.6%. There was one subject with gastric cancer, distributed in OLGA 4 stage. The PGI levels of males were higher than those in females (*p* < 0.05, Table 2).

### 3.2. OLGA Stage

According to the OLGA stage system, there were 176 (38.85%) subjects in Stage 0, 81 (17.88%) in Stage 1, 126 (27.81%) in Stage 2, 47 (10.38%) in Stage 3, and 23 (5.08%) in Stage 4 (Table 3). There were no significant differences in Hp infection prevalence among subjects with different OLGA stages. The smoking history showed statistical significance among different stages (*p* = 0.036). As OLGA stage increased, serum PGI, PGII, and PGR levels were significantly decreased (*p* < 0.01), while no significance was found between G-17 and OLGA stage.

### 3.3. HP Infection

Compared with the Hp-negative group, serum PGI, PGII, and G-17 levels were significantly elevated in the Hp-positive group, while PGR levels were significantly decreased (*p* < 0.01, Table 4).

Within the Hp-positive group, levels of serum PGI and PGR were clearly reduced with increased OLGA stage (*p* < 0.01, Table 5). The same pattern was found among the Hp-negative patients (p < 0.01). Although PGII levels decreased gradually with increased OLGA stage in the Hp-positive group (*p* < 0.05), there was no difference in the negative group. There was no statistically significant difference in G-17, both in Hp-positive and Hp-negative groups.

### 3.4. OLGA Stage Distribution by Age

Participants were divided into 22-44, 45-50, 51-57, and 58-80 age groups according to their number and gender. The OLGA stage increased with age in the Hp-positive group although subjects had no symptoms (*p* < 0.05, Table 6). However, this difference was not significant in the Hp-negative group.

Table 7 shows different serum PG levels in each age group. The infection rate of Hp showed no differences among the four age groups (*p* > 0.05). As the age increased, levels of serum PGI, PGII, and G-17 increased, whereas PGR was not obviously affected.

## 4. Discussion

There is a significant difference in the survival rate between those diagnosed with early GC and advanced GC [11]; the reason for this is a lack of effective treatments for advanced GC. AG and IM are recognized as precancerous lesions of GC, and Hp is a high-risk factor for GC [12–14]. It has been reported that a high-risk stage (defined as stage III or IV of the OLGA stage system) is closely related to the occurrence of GC [8, 12]. Therefore, detecting gastric mucosal atrophy and Hp infection in the general population is essential for early diagnosis.

Our study shows that the serum PG test is of similar value to OLGA stage in evaluating gastric mucosal atrophy in physical examination population from East China, especially PGI and PGR parameters. After more than 12 years of follow-up with 93 patients, Rugge et al. concluded that there was a significant inverse correlation between PGR and OLGA stage [15]. Dixon et al. and Wang et al. have also observed an obvious correlation between serum PG and OLGA stage in recent years, which is in line with our finding [10, 16].

Hokkanen et al. have reported that gender was the influencing factor of serum PG levels [17]. A previous population-based study conducted in North China shows men had higher normal PGI values than women [17]. In the present study, PGI levels of males were obviously higher than those of females, in agreement with results of previous studies. As expected, serum PG and G17 levels were significantly affected by age. A recent study has also reported that PGII levels increased while PGR declined with age in a healthy population [18, 19]. Hp prevalence of infection did not show a difference between males and females, similar to previous findings [20, 21].

It is generally recognized that Hp infection has an effect on serum PG levels. Hp is able to increase the gastrin level by stimulating G cells in the antrum, which directly stimulates the synthesis and secretion of PG in many cells, especially PG II [22]. In our study, the serum PG levels in the Hp-positive group were significantly different compared with the Hp-negative group. Ohkusa et al. have found that Hp infection upregulates the serum gastrin level, confirming that Hp infection could influence serum PG levels [23].

Interestingly, Hp infection was more common in subjects younger than 50 (45-50 range), accompanied by a lower risk OLGA stage. A previous study carried out by Nam et al. in a Korean population showed that Hp infection was most common in people aged 40 years or above, and there were no subjects younger than 30 in the high-risk stage [24]. Therefore, they concluded that the most suitable age for eradication of Hp may be under 30 years, before OLGA stage increased. In addition, Nam et al. proposed that early eradication of Hp can prevent most gastric cancer in patients with a low-risk OLGA stage, and surveillance endoscopic examination may not be necessary.

Another study conducted by Rugge et al. proposed that once Hp infection is detected, mandatory eradication treatment is required. Their evidence supported the view that the more severe the gastric disease is at the time of Hp eradication, the less reversible are the already established mucosal lesions will be [24]. Eradication before gastric atrophy provides the best opportunity to minimize risk, but beneficial effects of treatment still exist at an advanced age and in advanced disease. Even in patients with GC, eradication seems to at least delay the recurrence of cancer.

Our study shows that Hp infection is more prevalent among people under 50. Therefore, it is necessary to screen for Hp in China at less than 50 years of age, with a view to early diagnosis and effective treatment. With timely treatment, the gastric mucosa can be prevented from further developing precancerous lesions such as atrophy and IM which ultimately result in GC. The correlation between OLGA stage and age was also observed in Hp-positive participants. While PGI levels decreased with age, the OLGA scores were significantly increased in the Hp-positive group, similar to other research findings [25–27]. Hence, a regular endoscopy examination may be needed for elderly people.

The incidence of GC has obvious family aggregation and regional difference. Compared to South China, the GC incidence is significantly higher in eastern and northwest China. Common features of these areas are prolonged consumption of salted foods such as meat, salted fish, pickled vegetables, and seafood. In the present study, the prevalence of high-risk stages is high, perhaps because of the region's dietary habits.

The current study has several limitations. Firstly, the cross-sectional nature of the study makes it difficult to infer development of gastric mucosal atrophy and IM. Secondly, serum PG is affected by many factors, such as history of gastric acid suppression or Hp eradication, which has not been taken into account in our study. Thirdly, since the present study was based on one hospital and our research subjects were living in East China, our results may not represent the entire Chinese population. In addition, the sample size we have studied is limited and it would be prudent to carry out multicenter studies throughout China in the future.

In conclusion, significant correlations between PGI, PGR, and OLGA stage were observed among population from East China undergoing physical examination. Serum PG and OLGA stage showed consistent utility in the assessment of gastric mucosal atrophy. In addition, while early Hp eradication can delay the development of gastric into precancerous lesions, Hp screening is of great value in Chinese populations, and GC surveillance is necessary for the elderly. To better evaluate GC risk, we recommend combining serum PG with OLGA stage in clinical assessments.

## Figures and Tables

**Table 1:** The baseline characteristics of 453 subjects involved in the study.

Age, mean ± sd	52.46 ± 10.30
Gender (male), *n* (%)	216 (47.68)
BMI, mean ± sd	23.89 ± 3.29
Hp, *n* (%)	242 (53.42)
Smoking, *n* (%)	108 (23.84)
Alcohol consumption, *n* (%)	170 (37.53)
Family history of GC, *n* (%)	121 (26.7)
Atrophy, *n* (%)	230 (50.77)
Metathesis, *n* (%)	220 (48.56)
Dysplasia, *n* (%)	166 (36.64)
GC, *n* (%)	1 (0.2)

**Table 2:** Serum PG and pathology based on gender.

Group	Male	Female	*p* value
*N*	253	200	
PG I (*μ*g/L)	118.09 ± 74.06	107.31 ± 78.10	0.022
PG II (*μ*g/L)	13.09 ± 12.33	12.34 ± 12.68	0.656
PGR	9.95 ± 6.52	8.38 ± 6.60	0.061
G17 (pmol/L)	2.32 ± 6.38	3.48 ± 7.20	0.129

**Table 3:** The characteristics and serum PG in OLGA staging.

Group	Stage 0	Stage 1	Stage 2	Stage 3	Stage 4	*p* value
*N*	176	81	126	47	23	
Hp positive, *n* (%)	101 (57.38)	39 (48.15)	70 (55.56)	18 (38.30)	14 (60.87)	0.132
Smoking, *n* (%)	37 (21.02)	30 (37.04)	29 (23.02)	8 (17.02)	4 (17.39)	0.036
PG I (*μ*g/L)	123.18 ± 69.70	169.39 ± 74.36	114.20 ± 40.27	70.86 ± 26.86	53.35 ± 29.51	<0.01
PG II (*μ*g/L)	14.07 ± 11.84	14.73 ± 16.00	12.46 ± 10.50	9.41 ± 6.81	12.11 ± 12.22	<0.01
PGR	10.78 ± 6.77	12.19 ± 7.72	10.66 ± 4.91	8.79 ± 4.14	4.72 ± 2.61	<0.01
G-17 (pmol/L)	2.52 ± 7.91	2.50 ± 7.46	2.76 ± 4.42	1.69 ± 6.29	4.27 ± 15.32	0.702

**Table 4:** The baseline characteristics, serum PG levels, and OLGA staging distribution according to Hp status.

Group	Hp-positive	Hp-negative	*p* value
N	242	211	
PG I (*μ*g/L)	125.51 ± 66.46	99.72 ± 80.54	<0.01
PG II (*μ*g/L)	15.53 ± 10.51	8.59 ± 8.59	<0.01
PGR	7.54 ± 4.17	12.16 ± 6.99	<0.01
G-17 (pmol/L)	4.47 ± 8.38	1.48 ± 3.09	<0.01

**Table 5:** The OLGA stage distribution according to Hp status.

Group	Stage 0		Stage 1		Stage 2		Stage 3		Stage 4	
	HP (+)	HP (-)	HP (+)	HP (-)	HP (+)	HP (-)	HP (+)	HP (-)	HP (+)	HP (-)
*N*	101	75	39	42	70	56	18	29	14	9
PG I (*μ*g/L)	133.00 ± 68.27	111.00 ± 85.20	175.00 ± 58.18	150.50 ± 120.86	121.43 ± 35.72	92.06 ± 52.22	82.60 ± 32.73	66.98 ± 24.52	55.42 ± 26.19^∗^	52.00 ± 22.91^∗^
PG II (*μ*g/L)	15.93 ± 9.47	9.06 ± 11.00	20.60 ± 19.00	9.50 ± 11.43	15.00 ± 8.73	7.15 ± 7.58	10.73 ± 7.14	8.46 ± 5.30	15.72 ± 13.87^#^	10.00±5.66^∗∗^
PGR	7.62 ± 3.53	12.65 ± 7.62	9.82 ± 6.35	13.54 ± 5.44	8.07 ± 4.26	12.10 ± 5.68	6.65 ± 2.14	8.55 ± 6.31	4.32 ± 2.16^∗^	6.17 ± 1.22^∗^
G-17 (pmol/L)	5.18 ± 8.97	1.09 ± 2.12	5.63 ± 12.30	1.79 ± 2.95	4.05 ± 4.94	1.79 ± 3.22	4.35 ± 10.34	1.32 ± 6.23	5.18±15.94^∗∗^	4.27±15.14^∗∗^

^∗^ *p* < 0.01, compared with the different OLGA stages in the same Hp group; ^#^*p* < 0.05, compared with the different OLGA stages in the same Hp group; ^∗∗^*p* > 0.05, compared with the different OLGA stages in the same Hp group.

**Table 6:** The OLGA stage according to age intervals.

OLGA stage, *n* (%)	Age				
	22-44	45-50	51-57	58-80	*p* value
Total (*n* = 453)	109	122	102	120	
0	53 (48.62)	44 (36.06)	37 (36.24)	42 (35.00)	0.103
1	14 (12.84)	24 (19.67)	13 (12.75)	30 (25.00)	
2	30 (27.52)	37 (30.33)	31 (30.39)	28 (23.33)	
3	8 (7.34)	10 (8.20)	17 (16.67)	12 (10.00)	
4	4 (3.69)	7(5.74)	4(3.95)	8(6.67)	
Hp+ (*n* = 241)	61	73	44	63	
0	33 (54.10)	29 (39.73)	17 (38.64)	22 (34.92)	<0.01
1	5 (8.20)	15 (20.55)	3 (6.82)	16 (25.40)	
2	19 (31.15)	22 (30.14)	15 (34.10)	14 (22.22)	
3	2 (3.27)	4 (5.48)	8 (18.18)	4 (6.34)	
4	3 (3.28)	3 (4.10)	1 (2.26)	7 (11.11)	
Hp- (*n* = 212)	48	49	58	57	
0	20 (41.67)	15 (30.61)	20 (34.48)	20 (35.10)	0.923
1	9 (18.75)	9 (18.37)	10 (17.24)	14 (24.56)	
2	11 (22.92)	15 (30.61)	16 (27.59)	14 (24.56)	
3	6 (12.5)	6 (12.24)	9 (15.51)	8 (14.04)	
4	1 (4.21)	4 (8.17)	3 (5.18)	1 (1.74)	

**Table 7:** The serum test according to age intervals.

Group	Age				
	22-44	45-50	51-57	58-80	*p* value
Hp, *n* (%)	61(55.96)	73(59.83)	44(43.13)	63(52.50)	0.013^∗^
PG I (*μ*g/L)	102.5 ± 70.38	109.44 ± 83.28	114.53 ± 65.04	136.48 ± 102.6	<0.01
PG II (*μ*g/L)	12.96 ± 11.51	12.09 ± 10.65	12.03 ± 10.96	15.20 ± 13.32	0.014
PGR	8.97 ± 6.28	10.03 ± 6.18	9.44 ± 6.53	8.60 ± 7.35	0.731
G17 (pmol/L)	2.61 ± 6.29	3.88 ± 8.50	1.77 ± 4.33	2.52 ± 7.97	0.021

^∗^ *p* = 0.013, range 45-50 compared to range 51-57.

## Data Availability

The retrospective data used to support the findings of this study are included within the supplementary information file.

## Supplementary Materials

Supplementary Materials The supplementary materials is the original data of the manuscript, including serum PG and G17 levels, HP status, and the history of smoking and drinking.
