# Supplementary material for: Pepsinogen Serology and Gastritis OLGA Staging in Mucosal Atrophy Assessment: A Cross-Sectional Study Involving East China Endoscopy Population
Source: Gastroenterol Res Pract. 2020 Apr 13;2020:2324505. doi: 10.1155/2020/2324505 (PMC7174926; doi:10.1155/2020/2324505)
Supplement: Supplementary Materials — The supplementary materials is the original data of the manuscript, including serum PG and G17 levels, HP status, and the history of smoking and drinking. [file 2324505.f1.pdf]

Numb	BMI	Gender (male,1;female,2)	Age	Smoking history (Yes,1;No,2)	Alcohol History (Yes,1;No,2)	GC family history (Yes,1;No,2)
Data001	23.67	1	55	2	2	1
Data002	23.67	1	49	1	1	1
Data003	23.03	1	52	2	1	1
Data004	23.44	2	55	1	2	2
Data005	32.65	1	44	2	1	1
Data006	29.41	1	40	2	2	2
Data007	21.97	1	58	1	2	1
Data008	18.87	2	47	2	2	2
Data009	17.09	2	56	1	2	2
Data010	29.59	1	66	2	2	1
Data011	23.32	1	76	2	1	2
Data012	26.27	1	67	2	1	1
Data013	24.46	2	48	2	2	2
Data014	18.59	1	56	2	1	1
Data015	28.38	2	51	2	2	2
Data016	26.49	2	52	2	2	1
Data017	26.08	1	62	2	1	2
Data018	22.99	1	57	1	1	1
Data019	23.44	2	70	1	2	2
Data020	22.49	1	27	2	1	1
Data021	25.18	1	36	2	1	2
Data022	35.69	1	36	2	2	2
Data023	29.07	1	44	2	1	1
Data024	22.49	1	45	1	1	1
Data025	23.5	2	47	2	2	2
Data026	34.11	2	49	1	2	2
Data027	31.16	2	50	1	2	2
Data028	21.88	1	52	2	2	1
Data029	24.91	2	56	2	2	2
Data030	21.63	1	32	1	2	1
Data031	29.74	1	42	2	1	1
Data032	22.32	2	42	2	2	1
Data033	26.67	2	45	1	2	2
Data034	21.05	1	46	1	2	1
Data035	20.45	2	50	2	2	2
Data036	25.95	1	52	1	1	1
Data037	22.49	1	55	2	2	1
Data038	20.94	2	61	1	2	2
Data039	22.66	2	63	2	2	2
Data040	23.67	1	55	2	1	1
Data041	29.53	2	50	2	2	2

Data042	23.51	1	51	2	2	2
Data043	30.4	1	45	2	2	1
Data044	27.68	1	72	2	2	2
Data045	23.12	1	40	2	1	1
Data046	24.89	2	49	2	2	2
Data047	25.31	2	63	2	2	2
Data048	29.53	2	43	2	2	2
Data049	22.31	2	60	2	2	2
Data050	23.5	2	80	2	2	1
Data051	20.4	2	64	2	2	2
Data052	29.73	1	67	1	2	2
Data053	21.8	1	65	1	1	2
Data054	24.16	1	51	2	2	1
Data055	25.22	1	64	2	2	2
Data056	24.02	1	45	2	1	1
Data057	27.68	1	51	2	2	1
Data058	26.3	1	52	2	1	1
Data059	22.6	1	61	2	2	1
Data060	22.06	2	42	2	2	2
Data061		1	46	1	1	2
Data062	24.51	1	46	2	2	1
Data063	20.03	2	47	2	2	2
Data064	22.89	2	53	2	2	1
Data065	22.86	1	63	1	1	1
Data066	20.2	1	71	2	2	2
Data067	28.69	2	60	2	2	2
Data068	26.73	1	43	1	2	1
Data069	23.67	1	46	2	2	2
Data070	25.06	1	48	2	2	1
Data071	29.78	2	49	2	2	2
Data072	27.34	2	50	2	2	2
Data073	26.89	1	51	1	2	1
Data074	26.67	2	51	2	2	2
Data075	22.04	1	69	2	1	2
Data076	21.51	1	74	2	2	2
Data077	22.04	2	60	1	2	2
Data078	24.24	2	35	2	2	2
Data079	25.97	1.00	41	1	1	2
Data080	26.45	1	52	2	1	1
Data081	21.48	2	53	2	2	2
Data082	24.49	1	37	2	2	2
Data083	29.88	1	38	1	2	1
Data084	21.19	2	27	2	2	2

Data085	22.19	2	41	2	2	2
Data086	24.61	2	46	2	2	2
Data087	28.65	1	47	2	1	2
Data088	30.82	2	48	1	2	2
Data089	18.29	2	61	1	2	1
Data090	22.64	2	62	2	2	2
Data091	19.59	1	44	1	2	2
Data092	22.04	2	58	1	1	1
Data093	23.3	2	40	2	2	2
Data094	26.79	1	40	1	1	2
Data095		1	52	1	2	2
Data096		1	37	1	1	2
Data097	17.63	1	25	1	2	2
Data098	22.6	2	70	2	2	2
Data099		1	57	1	1	2
Data100	19.79	2	47	2	2	2
Data101	20.6	2	47	2	2	2
Data102		1	51	1	1	2
Data103	23.96	1	43	1	1	2
Data104	19.31	2	38	2	2	2
Data105	21.44	2	46	2	2	2
Data106	23.23	2	48	2	2	1
Data107	27.83	1	50	2	2	2
Data108	23.26	1	52	2	2	2
Data109	21.65	1	57	2	2	2
Data110	29.79	2	53	2	2	2
Data111	24	1	72	1	1	2
Data112		2	60	2	2	1
Data113	28.18	2	64	2	2	2
Data114	29	2	65	2	2	2
Data115	23.98	1	60	1	1	2
Data116	21	1	51	1	1	2
Data117	24.8	1	47	2	2	1
Data118	29	1	38	2	2	1
Data119	22.64	1	43	1	1	2
Data120	21.91	2	29	2	2	2
Data121	20.76	1	41	1	2	2
Data122	20	1	35	2	2	2
Data123	30.23	1	36	1	1	2
Data124	24.19	1	40	2	2	2
Data125	23.85	1	41	2	2	2
Data126		1.00	44	1	1	2
Data127	16.22	2	30	2	2	2
Data128	22.14	2	30	2	2	2

Data129	25.48	2	31	1	2	2
Data130		1	41	1	1	2
Data131	24.12	2	36	2	2	2
Data132	24.47	2	37	2		2
Data133		2	40	2	2	1
Data134	28.43	2	45	2	2	1
Data135	20.28	2	45	2	2	2
Data136		1	46	1	1	2
Data137	22.89	2	46	1	2	2
Data138	24.17	1	49	2	2	2
Data139	26.06	2	50	2	2	2
Data140	26.37	2	51	2	2	2
Data141	25.21	2	54	2	2	2
Data142	30.32	2	57	2	2	2
Data143	28.6	1	52	1	1	2
Data144		2	61	2	2	2
Data145		2.00	66	2	2	2
Data146	25	1	60	1	1	2
Data147	24.21	1	65	1	1	2
Data148	21.67	2	29	1	2	2
Data149	24.13	2	38	2	2	1
Data150	22.35	2	40	2	2	2
Data151		2	40	2	2	2
Data152	22.07	2	44	2	2	2
Data153	28.9	1	37	2	2	2
Data154		1	41	1	1	1
Data155		2	28	2	2	2
Data156	22.01	2	39	2	2	2
Data157	17.9	1	46	1	1	2
Data158	20.48	1	47	2	1	1
Data159	27.2	2	48	2	2	2
Data160	22.97	1	50	1	1	2
Data161	24.22	1	51	2	2	1
Data162	26.27	1	55	1	1	2
Data163	22.66	2	52	2	2	2
Data164	25.16	2	52	2	2	2
Data165	22.27	2	58	2	2	2
Data166	22.41	2	63	2	2	1
Data167	24.22	2	71	2	2	2
Data168	21.44	2	70	2	2	2
Data169	24.22	2	55	2	2	2
Data170		1	42	2	2	2
Data171	20.44	1	44	2	1	2
Data172	23.14	2	46	1	2	2

Data173	25.71	1	70	2	1	1
Data174	22.27	2	48	2	1	2
Data175	29.04	1	50	2	2	1
Data176	24.31	2	61	2	2	2
Data177	21.38	1	74	1	1	1
Data178	24.61	1	47	1	2	2
Data179	25.71	1	61	1	1	2
Data180	22.65	1	38	2	2	1
Data181	23.51	1	48	1	2	2
Data182	23.66	1	49	2	1	2
Data183	26.26	2	50	1	2	1
Data184	19.63	2	49	2	2	2
Data185	20.83	2	53	1	2	2
Data186	23.66	1	61	2	1	1
Data187	25.97	2	61	2	2	2
Data188	34.16	1	60	1	2	2
Data189	18.37	2	70	2	2	2
Data190	29.67	1	46	1	2	2
Data191	20.76	1	50	1	1	2
Data192	19.82	1	53	1	2	2
Data193	28.41	1	55	2	1	1
Data194	27.68	1	44	1	2	1
Data195	24.22	1	43	2	1	1
Data196	20.96	2	44	2	2	2
Data197	20.9	1	58	1	1	1
Data198	24.14	2	63	1	2	2
Data199	23.44	2	61	1	2	2
Data200	19.29	2	62	2	2	2
Data201	17.3	1	65	1	2	1
Data202	25.83	1	66	1	1	1
Data203	24.09	2	70	1	2	2
Data204	22.76	1	53	1	2	1
Data205	24.96	1	46	2	1	2
Data206	23.83	1	47	1	1	1
Data207	20.31	1	55	2	2	1
Data208	24.22	1	55	1	2	2
Data209	22.06	1	47	1	2	2
Data210	20.52	1	51	2	1	1
Data211	23.63	1	58	1	1	1
Data212	18.67	2	64	2	2	2
Data213	19.26	1	44	2	2	1
Data214	27.89	2	61	2	2	2
Data215	23.03	1	50	1	1	1
Data216	23.05	2	50	1	2	2

Data217	18.67	2	45	2	2	2
Data218	23.67	1	80	2	1	2
Data219	19.14	2	69	2	2	2
Data220	20.89	2	76	2	2	1
Data221	24.73	1	46	2	2	1
Data222	25.06	1	49	2	1	1
Data223	21.94	2	34	2	2	2
Data224	22.65	1	52	1	2	2
Data225	22.41	1	76	1	1	2
Data226		1	46	2	2	
Data227	20.6	2	53	2	2	2
Data228	23.52	2	52	2	2	2
Data229	25.1	1	58	1	1	2
Data230	24.14	1	62	2	2	2
Data231	22.52	1	60	1	1	2
Data232	25.95	2	60	2	2	2
Data233	22	1	39	2	2	1
Data234	21.03	2	43	1	1	2
Data235	19.98	1	35	2	2	2
Data236		1	64	1	1	2
Data237	26.11	1	35	2	2	2
Data238	30.44	2	49	2	2	2
Data239	26.92	2	37	1	1	2
Data240	26.56	2	48	2	1	2
Data241	23.45	1	50	2	2	1
Data242	24.78	2	51	2	2	2
Data243	23.5	2	60	1	1	2
Data244	25.83	1	26	1	1	2
Data245		2	41	2	2	2
Data246	22.85	2	36	2	2	2
Data247	23.69	1	46	1	1	2
Data248	22.3	1	52	2	2	2
Data249	17.98	1	54	1	1	2
Data250	29.37	1	53	1	1	2
Data251	19.73	2	66	2	2	2
Data252	20.12	2	64	2	2	2
Data253	21.19	1	65	2	2	2
Data254	21.51	1	69	1	2	1
Data255	23.6	2	46	2	2	2
Data256	19.65	2	53	2	2	2
Data257	29.75	1	49	2	1	1
Data258	22.15	1	72	2	2	1
Data259	23.39	1	66	1	2	1
Data260	21.88	1	53	2	1	2

Data261	21.34	2	53	2	2	1
Data262	23.01	2	56	1	2	2
Data263	22.77	2	47	1	2	2
Data264	21.97	1	40	1	2	2
Data265	26.58	1	54	1	1	1
Data266	17.53	2	54	1	2	2
Data267	22.49	1	70	2	1	2
Data268	20.2	2	42	2	2	2
Data269	21.09	2	53	1	2	2
Data270	27.56	2	64	2	2	2
Data271	24.03	2	51	2	2	2
Data272	21.72	1	42	2	1	2
Data273	28.67	2	36	2	2	2
Data274	23.44	2	49	2	2	2
Data275	22.06	2	50	2	2	2
Data276	21.97	1	54	2	1	1
Data277	24.22	1	42	2	2	1
Data278	20.28	2	62	2	2	1
Data279	26.95	2	41	2	2	2
Data280	22.91	1	45	2	1	2
Data281	23.31	2	52	1	2	2
Data282	23.83	2	54	2	2	2
Data283	21.5	1	59	2	1	2
Data284	21.8	1	62	2	2	2
Data285	30.06	2	62	2	2	2
Data286	22.51	2	64	2	2	2
Data287	26.67	2	50	2	2	2
Data288	22.86	1	54	2	1	1
Data289	20.52	1	41	2	1	1
Data290	21.64	2	60	1	2	2
Data291	22.66	2	45	2	2	2
Data292	25.71	2	61	1	2	1
Data293	21.51	1	44	2	1	1
Data294	26.85	1	63	1	2	1
Data295	27.77	1	43	2	1	2
Data296	24.91	1	58	2	1	2
Data297	18.37	2	74	1	2	2
Data298	22.04	2	40	1	2	2
Data299	20.08	2	46	2	2	2
Data300	20.76	1	48	2	1	1
Data301	22.6	2	39	2	2	1
Data302	26.95	1	47	2	1	1
Data303	25.53	1	46	2	1	1
Data304	22.04	1	48	2	1	1

Data305	20.2	1	54	2	1	1
Data306	24.98	1	58	1	1	1
Data307		1	43	2	2	1
Data308		1	47	2	2	2
Data309	23.27	1	53	1	1	2
Data310	28.54	1	29	2	2	2
Data311	26.7	1	53	2	1	1
Data312	26.12	1	75	2	2	2
Data313	24	1	22	2	2	2
Data314	25.33	1	37	1	1	2
Data315	20	2	44	2	2	2
Data316	27.67	2.00	42	2	2	2
Data317	22.27	2	40	1	2	2
Data318	23.65	1	44	2	2	2
Data319	21.91	2.00	44	2	2	2
Data320	24.67	1	43	1	1	2
Data321	29.04	1	39	2	2	2
Data322		1	45	1	1	2
Data323	31.24	1	45	1	1	2
Data324	25.64	1	45	2	2	1
Data325	22.51	1	46	1	1	2
Data326	22.51	1	49	1	1	2
Data327	24.93	1	49	1	1	2
Data328	27.96	1	49	1	1	2
Data329	23.54	1	49	2	1	2
Data330	24.12	1	53	1	1	2
Data331		2	53	2	2	2
Data332	26.6	2	54	1	1	2
Data333	26.67	1	58	1	1	2
Data334		2	64	2	1	2
Data335	21.92	2	61	2	2	2
Data336	24.3	1	46	1	1	2
Data337		2	46	2	2	1
Data338		1.00	47	1	1	2
Data339	21.66	2	47	2	2	2
Data340	21.16	2	47	2	2	2
Data341	27.36	1	51	2	2	2
Data342	28.72	1	56	2	2	2
Data343	21.05	1	54	1	2	1
Data344	24.66	1	63	1	1	2
Data345	22.71	1	58	2	1	2
Data346	25.5	2	61	2	2	1
Data347	24.24	1	68	1	1	2
Data348	28.73	1	51	2	1	2

Data349	26.8	1	48	2	2	2
Data350	23.41	1	44	2	2	2
Data351	21.71	2	43	2	2	2
Data352	18.7	2	46	2	2	2
Data353	25.73	1	49	2	2	2
Data354	22.76	1	57	1	1	2
Data355	25.79	1	54	1	1	1
Data356	23.61	1	54	1	1	2
Data357	24.44	1	55	2	2	1
Data358	27.55	2	55	1	2	2
Data359	20.86	2	52	2	2	2
Data360		1	63	1	1	2
Data361	22.9	1	59	1	2	1
Data362	26.4	1	44	2	2	2
Data363	21.12	1	36	2	2	2
Data364	23.44	2	40	1	2	2
Data365	21.81	2	42	2	2	2
Data366	21.22	1	36	1	1	2
Data367	21.28	2	45	2	2	2
Data368		2	46	2	2	2
Data369		1	47	1	2	2
Data370		1	49	1	2	2
Data371		1	53	1	1	2
Data372	27.88	1	57	1	1	2
Data373	22.5	1	52	2	1	1
Data374	30.16	1	52	2	1	2
Data375	22.19	2	51	1	2	1
Data376	26.5	1	51	1	1	2
Data377		1	57	2	2	2
Data378	24.03	2	58	1	2	2
Data379		2	64	1	1	1
Data380	21.96	1	53	1	1	2
Data381	20.76	1	53	2	2	1
Data382	22.21	2	61	2	2	2
Data383	24.61	2	40	2	2	2
Data384	20.81	2	68	1	2	2
Data385	29.76	1	52	2	1	1
Data386	19.53	2	44	1	2	2
Data387	24.22	1	54	2	2	1
Data388	23.42	2	54	2	2	2
Data389	20.52	1	61	2	2	2
Data390	25.28	1	52	2	1	2
Data391	25.5	1	65	1	1	1
Data392	20.98	1	48	2	1	1

Data393	31.93	2	51	2	2	2
Data394	24.77	1	54	2	1	1
Data395	24.03	2	59	2	2	2
Data396	22.03	2	66	1	2	2
Data397	25.04	1	46	2	2	2
Data398	24.22	1	53	1	1	2
Data399	21.72	2	77	2	2	2
Data400	18.79	1	56	2	1	1
Data401	19.59	1	44	2	1	2
Data402	22.89	2	64	2	2	2
Data403	25.96	1	42	2	1	1
Data404	23.14	1	63	2	1	1
Data405	24.9	1	54	2	2	2
Data406	21.76	1	36	1	1	2
Data407	23.21	2	45	1	1	1
Data408	27.44	2	50	2	2	2
Data409	27.12	1	53	2	2	2
Data410	23.94	2	53	2	2	2
Data411	23.74	2	46	1	1	1
Data412	22.21	1	56	1	1	2
Data413	26.2	1	51	1	1	2
Data414	30.01	1	52	2	2	2
Data415	29.93	1	62	2	2	2
Data416	16.22	1	57	1	1	2
Data417		1.00	36	1	1	2
Data418	22.96	2	43	2	2	2
Data419	21.08	2	50	2	2	2
Data420	20.85	2	51	2	2	2
Data421	24.7	1	62	1	1	2
Data422		1	34	2	1	2
Data423	29.77	1	35	1	1	1
Data424	20.56	2	46	2	1	2
Data425	21.91	2	47	2	2	2
Data426	26.12	1	48	1	1	2
Data427		1	50	1	1	2
Data428	21.9	2	55	2	2	2
Data429		2	64	2	2	2
Data430	23.93	2	62	2	2	2
Data431	21.05	1	66	2	1	1
Data432	18.81	1	78	2	2	2
Data433	20.08	2	47	2	2	2
Data434	22.19	1	72	2	1	2
Data435	20.58	2	42	2	2	2
Data436	21.51	2	48	2	2	1

Data437	25.39	1	53	1	2	1
Data438	23.62	1	77	2	2	2
Data439	20.45	2	51	2	2	2
Data440	19.42	2	47	2	2	2
Data441		2.00	49	2	2	2
Data442	28.4	1	51	1	1	1
Data443	21.5	2	54	2	2	2
Data444		1	42	1	1	1
Data445	25.71	1	48	2	1	1
Data446	23.01	2	42	2	2	2
Data447	28.34	1	64	1	1	2
Data448		2	61	2	2	2
Data449	20.03	2	39	2	2	2
Data450	26.04	1	47	1	1	2
Data451	25.96	2	49	2	2	2
Data452	24.71	2	61	2	2	2
Data453	23.34	2	62	2	2	1

PGI	PGII	PGR	G17	Hp infection (Yes,1;No,2)	Gastroscope	Pathology of gastric antral
136.00	8.80	15.45	0.10	2	Gastritis, DU	Inflammation
118.09	9.53	12.39	1.35	2	Gastritis	Inflammation
111.00	5.80	19.14	0.80	2	Gastritis	Inflammation
120.00	6.00	20.00	1.20	2	Gastritis	Inflammation
133.00	3.00	11.00	3.00	1	Gastritis	Inflammation
69.05	5.14	13.42	1.10	2	Gastritis	Inflammation
354.00	24.00	14.75	0.40	2	Gastritis	Inflammation
65.50	9.40	6.97	26.98	1	Gastritis	Inflammation
240.00	30.00	8.00	9.00	1	Gastritis	Inflammation
317.00	50.00	6.34	2.00	1	Gastritis	Inflammation
259.00	14.70	17.62	0.90	1	Gastritis	Inflammation
59.78	9.46	6.32	0.50	2	Gastritis	Inflammation
54.54	12.50	4.36	18.43	1	Gastritis	Inflammation
200.00	15.00	13.33	4.40	1	Gastritis	Inflammation
54.72	4.71	11.62	0.85	1	Gastritis	Inflammation
93.00	12.00	7.75	0.50	1	Gastritis	Inflammation
169.00	25.00	6.76	0.60	1	Gastritis	Inflammation
205.00	22.00	9.32	1.30	2	Gastritis	Inflammation
137.00	17.00	8.06	0.10	2	Gastritis	Inflammation
105.31	15.26	6.90	5.61	1	Gastritis	Inflammation
102.50	21.27	4.82	1.91	1	Gastritis	Inflammation
92.73	13.37	6.94	9.13	1	Gastritis	Inflammation
144.66	10.48	10.94	1.31	1	Gastritis	Inflammation
147.00	14.00	10.50	1.50	1	Gastritis	Inflammation
117.32	18.86	6.22	5.17	1	Gastritis	Inflammation
124.63	15.44	8.07	63.54	1	Gastritis	Inflammation
66.00	9.00	7.33	0.40	1	Gastritis	Inflammation
144.81	15.93	9.09	2.25	1	Gastritis	Inflammation
112.93	14.83	7.62	6.67	1	Gastritis	Inflammation
107.00	5.50	19.45	1.00	2	Gastritis	Inflammation
43.59	3.60	12.11	0.63	2	Gastritis	Inflammation
61.96	4.32	14.36	1.13	2	Gastritis	Inflammation
149.00	24.30	6.13	3.40	2	Gastritis	Inflammation
159.00	6.90	23.04	0.10	2	Gastritis	Inflammation
75.00	7.00	10.71	0.10	2	Gastritis	Inflammation
308.91	28.53	10.83	35.88	2	Gastritis	Inflammation
87.00	15.00	5.80	1.50	2	Gastritis	Inflammation
100.00	7.50	13.33	0.60	2	Gastritis	Inflammation
306.00	40.00	7.76	85.00	2	Gastritis	Inflammation
296.00	21.00	14.10	0.10	2	Gastritis	Inflammation
195.00	12.00	16.25	3.60	1	Gastritis	Inflammation

106.00	7.00	15.14	2.00	1	Gastritis	Inflammation
284.00	28.00	10.14	2.10	2	Gastritis	Inflammation
404.00	56.00	7.21	2.70	2	Gastritis	Inflammation
109.00	5.50	19.82	0.20	1	Gastritis	Inflammation
344.06	66.05	5.21	47.38	1	Gastritis	Inflammation
92.74	14.60	6.35	0.77	1	Gastritis	Inflammation
99.00	5.00	19.80	2.40	2	Gastritis	Inflammation
365.00	19.00	19.21	2.80	2	Gastritis	Inflammation
144.00	15.00	9.60	1.70	2	Gastritis	Inflammation
376.00	22.00	17.09	2.00	2	Gastritis	Inflammation
162.00	9.00	18.00	0.20	1	Gastritis	Inflammation
276.00	17.00	16.24	65.00	2	Gastritis	Inflammation
118.53	15.48	7.66	1.96	1	Compound ulcer，Gastritics	Inflammation
90.68	6.60	13.74	0.86	2	Gastritis	Inflammation
224.00	35.00	6.40	21.00	1	Gastritis	Inflammation
127.00	15.00	8.47	0.40	2	Gastritis	Inflammation
141.91	17.04	8.33	4.27	1	Gastritis	Inflammation
110.00	7.00	15.71	1.00	2	Gastritis	Inflammation
684.00	27.00	75.33	7.00	1	Gastritis	Inflammation
224.38	35.43	6.33	7.28	1	Gastritis	Inflammation
142.39	23.10	6.16	11.61	1	Gastritis	Inflammation
86.00	5.00	17.20	2.40	1	Gastritis	Inflammation
146.70	29.94	4.90	18.75	1	Gastritis	Inflammation
142.00	18.00	7.89	0.50	1	Gastritis	Inflammation
101.00	10.00	10.10	0.20	1	Gastritis	Inflammation
224.00	30.00	7.47	0.40	1	Gastritis	Inflammation
64.00	5.00	12.80	1.00	2	Gastritis	Inflammation
167.00	9.00	18.56	0.10	2	Gastritis	Inflammation
103.00	10.00	10.30	0.10	2	Gastritis	Inflammation
65.59	4.80	13.67	0.93	2	Gastritis	Inflammation
85.00	7.00	12.14	0.80	2	Gastritis	Inflammation
165.00	20.00	8.25	0.10	2	Gastritis	Inflammation
187.70	22.30	8.40	10.00	2	Gastritis	Inflammation
78.00	11.00	7.09	0.10	2	Gastritis	Inflammation
153.00	17.50	8.74	12.70	2	Gastritis	Inflammation
123.00	6.30	19.52	0.10	2	Gastritis	Inflammation
117.04	12.00	9.75	5.18	1	Gastritis	Inflammation
280.39	20.30	13.82	22.59	2	Gastritis	Inflammation
152.00	6.50	23.38	0.90	2	Gastritis	Inflammation
130.00	10.00	13.00	2.70	2	Gastritis	Inflammation
143.43	21.40	6.70	19.75	1	Gastritis	Inflammation
137.70	20.69	6.66	9.92	1	Gastritis	Inflammation
123.36	16.41	7.52	1.61	1	Gastritis	Inflammation

97.52	14.02	6.96	1.13	1	Gastritis	Inflammation
89.40	10.13	8.83	4.09	1	Gastritis	Inflammation
99.60	14.15	7.04	13.30	1	Gastritis	Inflammation
111.44	17.30	6.44	4.85	1	Gastritis	Inflammation
137.53	15.47	8.89	2.43	1	Gastritis	Inflammation
135.96	20.11	6.76	3.18	1	Gastritis	Inflammation
77.00	3.90	19.40	0.10	2	Gastritis	Inflammation
107.00	17.00	6.29	10.00	2	Gastritis	Inflammation
138.38	17.29	8.00	12.97	1	Gastritis, DU	Inflammation
100.41	11.11	9.04	0.96	2	DU	Inflammation
123.57	6.82	18.12	0.50	2	DU	Inflammation
137.20	14.12	9.72	2.18	1	DU	Inflammation
185.69	26.66	6.97	15.34	1	Gastritis, DU	Inflammation
214.00	38.00	5.63	21.00	1	Gastritis	Inflammation
138.31	14.64	9.45	2.98	1	Gastritis	Inflammation
162.64	17.97	9.05	11.96	1	Gastritis	Inflammation
78.03	9.05	8.62	1.32	2	Gastritis	Inflammation
115.06	9.43	12.20	1.24	2	Gastritis	Inflammation
119.46	22.78	5.24	2.94	1	Gastritis	Inflammation
337.98	63.23	5.35	44.46	1	Gastritis	Inflammation
210.08	54.84	3.83	11.93	1	Gastritis	Inflammation
66.33	16.12	4.11	10.48	1	Gastritis	Inflammation
327.97	29.10	11.27	11.10	1	Gastritis	Inflammation
140.14	19.08	7.35	7.13	1	Gastritis	Inflammation
113.99	25.38	4.49	9.71	1	Gastritis	Inflammation
117.20	17.38	6.74	8.68	1	Gastritis	Inflammation
96.88	7.04	13.75	0.95	1	Gastritis	Inflammation
108.68	18.94	5.74	4.04	1	Gastritis	Inflammation
68.83	2.88	23.93	1.49	1	Gastritis	Inflammation
193.42	26.88	7.19	14.04	1	Gastritis	Inflammation
149.00	20.89	7.13	17.05	1	Gastritis	Inflammation
100.31	10.00	10.03	1.83	2	Gastritis	Inflammation
244.00	40.00	6.10	1.80	1	Gastritis	Inflammation
87.29	5.92	14.75	0.95	2	Gastritis	Inflammation
95.18	13.77	6.91	1.76	1	GU	Inflammation
148.22	15.92	9.31	3.42	2	GU	Inflammation
270.00	14.00	19.29	0.50	1	Gastritis	Inflammation
113.80	19.30	5.90	3.31	1	Gastritis	Inflammation
108.02	12.50	8.64	6.24	1	Gastritis	Inflammation
60.81	10.25	5.93	1.84	1	Gastritis	Inflammation
195.83	42.84	4.57	44.92	1	Gastritis	Inflammation
160.72	21.07	7.63	8.96	1	Gastritis	Inflammation
234.29	19.75	11.87	6.69	1	Gastritis	Inflammation
82.24	12.47	6.60	2.14	1	Gastritis	Inflammation

173.00	21.00	8.24	1.00	1	Gastritis	Inflammation
130.38	13.02	10.01	7.36	1	Gastritis	Inflammation
71.06	13.74	5.17	8.00	1	Gastritis	Inflammation
162.67	17.67	9.21	7.40	1	Gastritis	Inflammation
201.52	21.31	9.46	17.25	1	Gastritis	Inflammation
65.57	5.75	11.40	1.79	1	Gastritis	Inflammation
132.19	12.77	10.35	5.87	1	Gastritis	Inflammation
102.97	12.71	8.11	8.03	1	Gastritis	Inflammation
165.41	35.78	4.62	3.91	1	Gastritis	Inflammation
117.39	17.43	6.74	6.01	1	Gastritis	Inflammation
162.00	10.70	15.14	0.10	1	Gastritis	Inflammation
118.26	27.55	4.29	4.10	1	Gastritis	Inflammation
106.03	22.51	4.71	61.34	1	Gastritis	Inflammation
136.47	14.52	9.40	6.52	1	Gastritis	Inflammation
135.27	11.83	11.43	5.90	1	Gastritis	Inflammation
97.77	12.21	8.01	3.46	1	Gastritis	Inflammation
189.75	21.19	8.96	46.47	1	Gastritis	Inflammation
105.55	11.88	8.89	1.38	1	Gastritis	Inflammation
233.89	28.32	8.26	7.38	1	Gastritis	Inflammation
58.53	9.05	6.47	0.84	2	Gastritis	Inflammation
70.00	10.00	7.00	8.90	2	Gastritis	Inflammation
64.40	4.95	13.01	1.68	2	Gastritis	Inflammation
78.81	6.40	12.31	0.81	2	Gastritis	Inflammation
43.57	7.87	5.54	1.09	2	Gastritis	Inflammation
87.29	5.92	14.75	0.95	2	Gastritis	Inflammation
131.94	8.54	15.44	1.52	2	Gastritis	Inflammation
107.38	12.80	8.39	14.96	2	Gastritis	Inflammation
71.40	4.72	15.13	2.61	2	Gastritis	Inflammation
164.01	14.62	11.22	3.73	2	Gastritis	Inflammation
132.97	7.96	16.70	0.76	2	Gastritis	Inflammation
76.99	10.58	7.28	2.63	2	Gastritis	Inflammation
250.82	50.38	4.98	19.93	2	Gastritis	Inflammation
60.95	5.70	10.68	0.45	2	Gastritis	Inflammation
57.13	4.52	12.65	1.07	2	Gastritis	Inflammation
93.00	5.00	18.60	0.10	2	Gastritis	Inflammation
99.07	4.79	20.81	0.92	2	Gastritis	Inflammation
155.33	9.06	6.11	0.58	2	Gastritis	Inflammation
265.00	14.00	18.93	7.50	2	Gastritis	Inflammation
204.00	10.50	19.43	3.80	2	Gastritis	Inflammation
207.00	21.00	9.86	20.00	2	Gastritis	Inflammation
101.00	7.20	14.03	0.10	2	Gastritis	Inflammation
106.18	15.68	6.77	9.48	1	Gastritis, DU	Inflammation
86.09	6.91	12.46	12.71	1	Gastritis, DU	Inflammation
106.00	5.70	18.60	4.80	2	Gastritis	Inflammation

83.00	11.00	7.55	21.00	1	Gastritis	Inflammation
80.16	26.49	3.03	13.53	1	Gastritis	Inflammation
103.00	18.00	5.72	10.00	1	Gastritis	Inflammation
190.00	12.80	14.84	0.60	1	Gastritis	Inflammation
186.00	12.30	15.12	2.60	2	Atrophic gastriyis	Inflammation
98.00	5.90	16.61	85.00	1	Atrophic gastriyis	Inflammation
348.00	30.00	11.60	0.10	1	Atrophic gastriyis	Atrophy
186.00	23.00	8.09	2.50	2	Atrophic gastriyis	Atrophy
235.00	18.50	12.70	24.00	2	Atrophic gastriyis	Atrophy
187.00	12.00	15.58	0.40	2	Atrophic gastriyis	Atrophy
319.62	37.44	8.54	22.73	2	Atrophic gastriyis	Atrophy
104.00	3.90	26.67	6.50	2	Atrophic gastriyis	Atrophy
156.00	10.50	14.86	1.70	2	Atrophic gastriyis	Atrophy
195.00	16.00	12.19	0.20	2	Atrophic gastriyis	Atrophy
50.00	3.60	13.89	1.70	2	Atrophic gastriyis	Atrophy
172.00	9.00	19.11	2.40	2	Atrophic gastriyis	Atrophy
201.00	17.00	11.82	7.90	1	Atrophic gastriyis	Atrophy
104.00	6.00	17.33	1.50	2	Atrophic gastriyis	Inflammation
125.00	10.50	11.90	4.60	1	Atrophic gastriyis	Atrophy
46.00	3.30	14.38	0.40	2	Atrophic gastriyis	Atrophy
238.00	20.00	11.90	25.00	2	Atrophic gastriyis	Atrophy
301.00	16.00	18.81	13.60	1	Atrophic gastriyis	Atrophy
346.00	75.00	4.61	10.50	1	Atrophic gastriyis	Atrophy
275.00	55.00	5.00	14.00	1	Atrophic gastriyis	Atrophy
152.00	7.00	21.71	2.60	1	Atrophic gastriyis	Atrophy
259.56	58.20	5.08	23.47	1	Atrophic gastriyis	Atrophy
171.00	8.70	19.66	0.30	1	Atrophic gastriyis	Atrophy
124.00	5.60	22.14	0.20	1	Atrophic gastriyis	Atrophy
203.00	15.80	12.85	0.70	2	Atrophic gastriyis	Atrophy
163.00	10.00	16.30	0.60	2	Atrophic gastriyis	Inflammation
234.00	28.00	8.36	1.10	1	Atrophic gastriyis	Atrophy
114.00	6.00	19.00	0.10	2	Atrophic gastriyis	Atrophy
301.00	17.00	17.71	3.00	2	Atrophic gastriyis	Atrophy
164.00	20.60	7.96	0.30	1	Atrophic gastriyis	Atrophy
195.00	16.00	12.19	1.00	1	Atrophic gastriyis	Atrophy
90.00	4.50	20.00	0.20	2	Atrophic gastriyis	Atrophy
146.86	14.95	9.82	2.80	1	complex ulcer	Atrophy
200.00	16.00	12.50	1.30	1	Atrophic gastriyis	Atrophy
175.00	25.00	7.00	1.60	1	Atrophic gastriyis	Atrophy
262.00	15.40	17.01	4.20	2	Atrophic gastriyis	Atrophy
181.00	16.00	11.31	1.30	2	Atrophic gastriyis	Atrophy
177.33	41.66	4.26	31.56	2	Atrophic gastriyis	Atrophy
105.00	5.30	19.81	0.10	2	Atrophic gastriyis	Atrophy
181.00	25.00	7.24	1.20	1	Atrophic gastriyis	Atrophy

159.89	35.99	4.44	19.47	1	Atrophic gastriyis	Atrophy
145.00	8.00	18.13	2.40	2	Atrophic gastriyis	Atrophy
231.00	23.00	10.04	0.10	1	Atrophic gastriyis	Atrophy
201.00	10.50	19.14	3.50	2	Atrophic gastriyis	Atrophy
62.57	2.67	23.44	1.45	2	Atrophic gastriyis	Atrophy
192.00	10.00	19.20	10.80	1	Atrophic gastriyis	Atrophy
147.13	14.73	9.99	5.63	1	Atrophic gastriyis	Atrophy
179.00	13.30	13.46	0.20	2	Atrophic gastriyis	Atrophy
138.00	20.00	6.90	20.00	1	Atrophic gastriyis	Inflammation
162.33	13.09	12.40	52.44	1	Atrophic gastriyis	Atrophy
180.52	34.45	5.24	3.60	1	Atrophic gastriyis	Atrophy
103.96	9.02	11.53	3.05	1	Atrophic gastriyis	Atrophy
207.75	58.54	3.55	39.65	1	Atrophic gastriyis	Atrophy
158.80	23.70	6.70	11.13	1	Atrophic gastriyis	Atrophy
311.31	50.76	6.13	25.56	1	Atrophic gastriyis	Atrophy
186.13	38.05	4.89	6.19	1	Atrophic gastriyis	Atrophy
110.46	7.17	15.42	2.33	2	Atrophic gastriyis	Atrophy
40.81	3.07	13.27	2.37	2	Atrophic gastriyis	Inflammation
76.90	6.60	11.66	0.41	2	Atrophic gastriyis	Atrophy
340.65	40.76	8.36	15.48	2	Atrophic gastriyis	Atrophy
60.37	4.74	12.73	2.47	2	Atrophic gastriyis,GU	Atrophy
259.00	10.00	25.90	0.10	1	Atrophic gastriyis,GU	Atrophy
170.60	22.27	7.66	5.92	1	Atrophic gastriyis,GU	Atrophy
84.47	5.53	15.27	3.85	1	Atrophic gastriyis	Atrophy
149.57	20.80	7.19	7.38	1	Atrophic gastriyis	Atrophy
204.98	33.31	6.15	4.89	1	Atrophic gastriyis	Atrophy
171.00	13.96	12.25	5.77	1	Atrophic gastriyis	Atrophy
55.17	3.89	14.18	1.23	2	Atrophic gastriyis	Atrophy
53.04	4.73	11.21	2.13	2	Atrophic gastriyis	Atrophy
58.51	4.85	12.06	0.82	2	Atrophic gastriyis	Atrophy
273.20	23.48	11.63	6.96	2	Atrophic gastriyis	Atrophy
71.85	6.02	11.94	1.08	2	Atrophic gastriyis	Atrophy
169.43	17.53	9.66	1.88	2	Atrophic gastriyis	Atrophy
62.35	4.68	13.33	0.74	2	Atrophic gastriyis	Atrophy
132.17	12.93	10.23	9.20	2	Atrophic gastriyis	Atrophy
117.61	8.64	13.61	1.63	2	Atrophic gastriyis	Atrophy
214.03	20.24	10.57	7.30	2	Atrophic gastriyis	Atrophy
169.00	22.00	7.68	2.10	1	Atrophic gastriyis	Atrophy
148.00	26.72	5.54	12.63	1	Atrophic gastriyis	Inflammation
105.00	7.00	15.00	1.10	2	Atrophic gastriyis	Atrophy
185.00	11.00	16.80	62.00	1	Atrophic gastriyis	Atrophy
96.00	6.90	13.91	0.60	2	Atrophic gastriyis	Atrophy
199.00	20.00	9.95	7.50	1	Atrophic gastriyis	Atrophy
138.00	15.00	9.20	0.10	1	Atrophic gastriyis	IM

142.00	15.00	9.47	0.50	1	Atrophic gastriyis	IM
137.00	17.00	8.06	8.40	1	Atrophic gastriyis	IM
79.06	6.89	11.47	0.70	2	Atrophic gastriyis	Dysplasia
171.08	32.93	5.20	1.37	1	Atrophic gastriyis	IM
133.00	15.00	8.87	0.50	1	Atrophic gastriyis	Dysplasia
160.00	13.70	11.68	0.10	1	Atrophic gastriyis	IM
131.00	11.60	11.29	1.30	2	Atrophic gastriyis	Atrophy
120.00	7.90	15.19	4.20	1	Atrophic gastriyis	IM
127.00	8.30	15.30	0.50	2	Atrophic gastriyis	IM
66.00	3.30	20.00	1.00	2	Atrophic gastriyis	Inflammation
122.82	29.13	4.22	1.14	1	Atrophic gastriyis	IM
96.45	6.32	15.25	0.81	2	Atrophic gastriyis	Dysplasia
44.58	3.27	13.62	0.73	2	Atrophic gastriyis	Dysplasia
72.41	2.59	28.00	5.07	2	Atrophic gastriyis	IM
86.00	6.70	12.84	0.10	2	Atrophic gastriyis	IM
70.00	6.50	10.77	4.80	2	Atrophic gastriyis	Dysplasia
131.53	7.50	4.07	68.38	1	Atrophic gastriyis	IM
56.83	73.50	4.21	75.49	1	Atrophic gastriyis	Dysplasia
170.00	17.00	10.00	0.10	2	Atrophic gastriyis	IM
75.85	5.86	12.94	1.17	2	Atrophic gastriyis	IM
72.78	7.64	9.53	1.12	2	Atrophic gastriyis	Inflammation
49.41	2.05	24.09	1.02	2	Atrophic gastriyis	IM
93.41	9.45	9.89	2.81	2	Atrophic gastriyis	IM
118.00	10.00	11.80	2.30	2	Atrophic gastriyis	Atrophy
63.96	3.42	18.69	1.11	2	Atrophic gastriyis	IM
100.00	12.00	8.33	1.60	2	Atrophic gastriyis	Dysplasia
94.00	8.00	11.75	0.90	1	Atrophic gastriyis	Atrophy
106.61	7.30	14.60	1.18	2	Atrophic gastriyis	Atrophy
196.00	30.00	6.53	14.00	1	Atrophic gastriyis	IM
121.85	34.08	3.57	3.57	1	Atrophic gastriyis	IM
77.65	5.52	14.06	1.00	2	Atrophic gastriyis	IM
147.53	15.57	9.48	6.84	1	Atrophic gastriyis,DU	IM
285.00	25.00	11.40	4.80	1	Atrophic gastriyis	IM
85.97	10.99	7.82	3.12	1	Atrophic gastriyis	IM
98.32	11.41	8.61	1.99	1	Atrophic gastriyis	Dysplasia
192.00	15.40	12.47	2.20	1	Atrophic gastriyis	IM
170.00	17.00	10.00	2.00	2	Atrophic gastriyis	Inflammation
60.00	6.00	12.00	0.20	1	Atrophic gastriyis	IM
107.07	11.03	9.26	5.13	1	Atrophic gastriyis	IM
115.00	8.00	14.38	0.10	1	Atrophic gastriyis	Inflammation
121.00	15.00	8.07	3.00	1	Atrophic gastriyis	Atrophy
100.00	14.51	6.89	1.80	1	Atrophic gastriyis	Dysplasia
200.00	19.00	10.53	4.00	1	Atrophic gastriyis	Atrophy
147.00	13.70	10.37	0.10	1	Atrophic gastriyis	Dysplasia

30.00	2.50	12.00	1.50	1	Atrophic gastriyis	Atrophy
133.00	21.00	6.33	2.70	1	Atrophic gastriyis	IM
126.02	13.43	9.38	4.32	1	Atrophic gastriyis,DU	Atrophy
108.60	7.99	13.59	8.42	1	Atrophic gastriyis,DU	IM
97.94	12.10	8.10	11.71	1	Atrophic gastriyis,DU	IM
126.97	14.16	8.97	4.50	2	Atrophic gastriyis,DU	Dysplasia
121.00	11.60	10.43	0.10	1	Atrophic gastriyis	Dysplasia
130.00	10.00	13.00	3.20	1	Atrophic gastriyis	IM
128.48	20.18	6.37	5.01	1	Atrophic gastriyis	IM
99.44	3.75	26.50	1.68	2	Atrophic gastriyis	IM
67.00	8.00	8.38	15.00	1	Atrophic gastriyis	IM
73.44	7.47	9.83	1.01	2	Atrophic gastriyis	IM
72.00	14.70	4.92	0.10	1	Atrophic gastriyis	IM
160.61	53.15	3.02	37.79	1	Atrophic gastriyis	IM
119.19	17.63	6.76	3.74	1	Atrophic gastriyis	Dysplasia
102.04	13.70	7.45	9.96	1	Atrophic gastriyis	Dysplasia
130.67	22.56	5.79	5.71	1	Atrophic gastriyis	Atrophy
124.74	14.94	8.35	4.10	1	Atrophic gastriyis	IM
97.42	7.67	12.70	3.17	1	Atrophic gastriyis	IM
113.58	19.65	5.78	5.47	1	Atrophic gastriyis	IM
103.26	17.27	5.98	5.80	1	Atrophic gastriyis	Atrophy
109.28	17.69	6.18	2.46	1	Atrophic gastriyis	Inflammation
112.79	14.74	7.65	7.00	1	Atrophic gastriyis	IM
101.90	20.50	5.08	4.28	1	Atrophic gastriyis	Atrophy
109.59	14.97	7.32	3.06	1	Atrophic gastriyis	Dysplasia
119.89	16.81	7.13	5.27	1	Atrophic gastriyis	Atrophy
145.25	21.94	6.62	2.41	1	Atrophic gastriyis	IM
115.23	20.30	5.68	11.40	1	Atrophic gastriyis	Dysplasia
120.88	12.45	9.71	5.77	1	Atrophic gastriyis	Atrophy
147.25	25.30	5.82	11.35	1	Atrophic gastriyis	IM
180.13	30.31	5.94	3.97	1	Atrophic gastriyis	IM
163.81	15.13	10.82	1.10	2	Atrophic gastriyis	IM
112.01	13.36	8.38	2.44	2	Atrophic gastriyis	IM
176.31	30.31	5.82	24.79	2	Atrophic gastriyis	IM
59.76	4.97	12.02	4.88	2	Atrophic gastriyis	IM
81.61	3.23	25.27	7.32	2	Atrophic gastriyis	IM
187.09	12.06	15.51	2.30	2	Atrophic gastriyis	IM
151.69	12.46	12.17	3.10	2	Atrophic gastriyis	Dysplasia
64.48	5.21	12.39	3.15	2	Atrophic gastriyis	Dysplasia
88.70	6.81	13.03	1.00	2	Atrophic gastriyis	IM
86.86	5.57	15.60	1.23	2	Atrophic gastriyis	IM
127.96	22.48	5.69	18.13	2	Atrophic gastriyis	IM
76.08	8.80	8.65	8.28	2	Atrophic gastriyis	IM
139.00	12.00	11.58	1.00	1	Atrophic gastriyis,GU	IM

97.80	7.14	13.69	2.28	2	Atrophic gastriyis	IM
90.34	8.08	11.18	2.32	1	Atrophic gastriyis	IM
110.28	12.54	8.79	5.10	1	Atrophic gastriyis	IM
114.52	14.73	7.77	5.77	1	Atrophic gastriyis	IM
183.06	22.20	8.25	8.90	1	Atrophic gastriyis	IM
71.05	3.20	22.20	2.70	1	Atrophic gastriyis	Inflammation
159.23	21.01	7.58	2.54	1	Atrophic gastriyis	IM
106.03	12.24	8.66	7.51	1	Atrophic gastriyis	Atrophy
138.06	13.69	10.09	4.54	1	Atrophic gastriyis	Atrophy
131.00	25.00	5.24	32.00	1	Atrophic gastriyis	Inflammation
144.14	19.54	7.38	5.08	1	Atrophic gastriyis	Dysplasia
118.46	32.00	3.70	3.22	1	Atrophic gastriyis	Dysplasia
145.13	19.59	7.41	32.04	1	Atrophic gastriyis	Dysplasia
40.29	2.52	16.01	5.30	2	Atrophic gastriyis	IM
65.98	3.33	19.84	1.01	2	Atrophic gastriyis	IM
100.00	8.00	12.50	8.90	2	Atrophic gastriyis	Dysplasia
57.47	3.01	19.07	2.36	2	Atrophic gastriyis	Dysplasia
204.86	24.32	8.42	2.66	2	Atrophic gastriyis	IM
82.22	7.15	11.50	1.75	2	Atrophic gastriyis	Atrophy
124.44	6.76	18.42	1.82	2	Atrophic gastriyis	Atrophy
74.01	6.29	11.77	0.86	2	Atrophic gastriyis	IM
148.98	14.99	9.94	9.75	2	Atrophic gastriyis	IM
55.54	4.72	11.76	0.94	2	Atrophic gastriyis	Atrophy
72.31	4.63	15.61	3.38	2	Atrophic gastriyis	IM
100.96	22.50	4.49	18.11	2	Atrophic gastriyis	IM
107.00	17.00	6.29	0.85	2	Atrophic gastriyis	IM
63.21	2.82	22.41	7.82	2	Atrophic gastriyis	Atrophy
85.89	7.25	11.85	2.20	2	Atrophic gastriyis	IM
112.70	12.73	8.85	11.18	2	Atrophic gastriyis	Atrophy
143.00	20.00	7.15	0.60	2	Atrophic gastriyis	IM
62.73	3.24	19.36	2.30	2	Atrophic gastriyis	Inflammation
124.59	10.99	11.33	1.42		Atrophic gastriyis	Atrophy
125.00	7.50	16.67	0.70	1	Atrophic gastriyis	Atrophy
131.00	30.00	4.37	0.10	1	Atrophic gastriyis	Atrophy
107.25	16.99	6.31	4.14	1	Atrophic gastriyis	IM
59.00	6.30	9.37	22.00	2	Atrophic gastriyis	IM
60.43	3.61	16.75	1.52	2	Atrophic gastriyis	Dysplasia
65.00	9.00	7.22	0.10	2	Atrophic gastriyis	Inflammation
90.00	15.00	6.00	0.10	1	Atrophic gastriyis	IM
69.32	11.17	6.13	5.47	2	Atrophic gastriyis	Dysplasia
115.00	22.00	5.23	10.00	2	Atrophic gastriyis	IM
48.20	5.64	8.55	0.80	2	Atrophic gastriyis	IM
60.00	7.00	8.57	0.10	2	Atrophic gastriyis	IM
60.50	10.40	5.80	18.50	1	Atrophic gastriyis	IM

66.98	8.70	7.70	0.81	2	Atrophic gastriyis	Dysplasia
126.30	17.36	7.28	1.20	1	Atrophic gastriyis	Atrophy
70.00	11.00	8.36	1.30	1	Atrophic gastriyis	Dysplasia
80.70	2.89	6.40	11.70	1	Atrophic gastriyis	Dysplasia
64.27	7.03	9.14	1.22	2	Atrophic gastriyis	Dysplasia
78.00	9.70	8.04	0.10	2	Atrophic gastriyis	IM
71.00	9.00	7.89	1.00	2	Atrophic gastriyis	IM
62.24	10.45	5.95	3.76	1	Atrophic gastriyis	Dysplasia
86.00	13.00	6.62	0.50	2	Atrophic gastriyis	IM
50.00	10.22	4.89	1.50	1	Atrophic gastriyis	IM
68.94	12.96	5.32	0.95	2	Atrophic gastriyis	Atrophy
104.00	6.00	17.33	18.00	2	Atrophic gastriyis	IM
147.69	25.23	5.85	5.06	1	Atrophic gastriyis	Dysplasia
65.73	3.70	17.74	7.24	2	Atrophic gastriyis	IM
85.58	7.97	10.74	4.93	1	Atrophic gastriyis	Dysplasia
68.34	6.85	9.98	3.08	1	Atrophic gastriyis	Atrophy
113.91	16.53	6.89	6.94	1	Atrophic gastriyis	IM
80.39	9.78	8.22	0.85	1	Atrophic gastriyis	IM
123.66	29.37	4.21	6.83	2	Atrophic gastriyis	IM
64.58	9.41	6.87	0.80	2	Atrophic gastriyis	Atrophy
62.64	4.41	14.22	2.54	2	Atrophic gastriyis	Dysplasia
62.16	4.01	15.51	1.20	2	Atrophic gastriyis	IM
87.61	10.15	8.63	2.32	2	Atrophic gastriyis	Dysplasia
183.36	39.76	4.61	17.97	1	GU	Dysplasia
95.09	13.58	7.00	5.40	1	Atrophic gastriyis	IM
85.76	18.07	4.75	11.77	1	Atrophic gastriyis	IM
63.25	10.07	6.28	15.38	1	Atrophic gastriyis	Dysplasia
84.50	11.78	7.17	1.68	1	Atrophic gastriyis	Dysplasia
71.31	9.04	7.89	1.40	1	Atrophic gastriyis	IM
92.55	24.99	3.70	35.33	2	Atrophic gastriyis	IM
76.14	5.76	13.23	0.95	2	Atrophic gastriyis	Dysplasia
53.66	2.22	24.20	1.69	2	Atrophic gastriyis	IM
63.06	4.90	12.87	1.03	2	Atrophic gastriyis	IM
101.36	8.46	11.99	0.72	2	Atrophic gastriyis	Dysplasia
62.41	6.19	10.08	8.22	2	Atrophic gastriyis	IM
37.38	5.09	7.34	4.39	2	Atrophic gastriyis	Dysplasia
70.86	8.98	7.89	37.53	2	Atrophic gastriyis	Dysplasia
89.50	13.65	6.56	1.32	2	Atrophic gastriyis	Dysplasia
51.00	11.00	4.64	2.70	1	Atrophic gastriyis	Dysplasia
63.36	17.77	3.56	0.10	1	Atrophic gastriyis	Dysplasia
45.69	7.41	6.17	19.40	2	Atrophic gastriyis	Dysplasia
87.31	28.93	3.02	17.28	1	Atrophic gastriyis	Dysplasia
63.00	9.47	6.64	1.26	1	Atrophic gastriyis	Dysplasia
56.97	12.07	4.72	16.53	1	Atrophic gastriyis	Dysplasia

52.00	10.00	5.20	0.10	2	Atrophic gastriyis	Dysplasia
80.26	17.62	4.56	18.05	1	Atrophic gastriyis	IM
76.00	13.00	5.85	0.10	2	Atrophic gastriyis	Dysplasia
54.85	12.11	4.53	9.52	1	Atrophic gastriyis	Dysplasia
105.10	25.57	4.11	3.02	1	Atrophic gastriyis	Dysplasia
55.99	20.87	2.68	55.90	1	Atrophic gastriyis	Dysplasia
148.99	25.95	5.74	13.74	2	Atrophic gastriyis	Dysplasia
35.71	13.81	2.59	1.58	1	Atrophic gastriyis,GU	IM
50.94	12.24	4.16	16.73	2	Atrophic gastriyis	Dysplasia
39.06	7.67	5.09	4.18	1	Atrophic gastriyis	Dysplasia
51.84	24.12	2.15	1.41	1	Atrophic gastriyis	Dysplasia
42.17	8.20	5.14	6.17	1	Atrophic gastriyis	Dysplasia
40.75	6.52	6.25	0.10	2	Atrophic gastriyis	Dysplasia
74.15	10.38	7.14	1.41	2	Atrophic gastriyis	Dysplasia
44.64	5.46	8.17	10.04	2	Atrophic gastriyis	Dysplasia
53.35	8.65	6.17	4.27	2	Atrophic gastriyis	Atrophy
38.27	27.06	1.41	58.78	1	Atrophic gastriyis	Dysplasia

Pathology of gastric body	OLGA stage
Inflammation	0
Inflammation	0
Inflammation	0
Inflammation	0
Inflammation	0
Inflammation	0
Inflammation	0
Inflammation	0
Inflammation	0
Inflammation	0
Inflammation	0
Inflammation	0
Inflammation	0
Inflammation	0
Inflammation	0
Inflammation	0
Inflammation	0
Inflammation	0
Inflammation	0
Inflammation	0
Inflammation	0
Inflammation	0
Inflammation	0
Inflammation	0
Inflammation	0
Inflammation	0
Inflammation	0
Inflammation	0
Inflammation	0
Inflammation	0
Inflammation	0
Inflammation	0
Inflammation	0
Inflammation	0
Inflammation	0
Inflammation	0
Inflammation	0
Inflammation	0
Inflammation	0
Inflammation	0
Inflammation	0

Inflammation	0
Inflammation	0
Inflammation	0
Inflammation	0
Inflammation	0
Inflammation	0
Inflammation	0
Inflammation	0
Inflammation	0
Inflammation	0
Inflammation	0
Inflammation	0
Inflammation	0
Inflammation	0
Inflammation	0
Inflammation	0
Inflammation	0
Inflammation	0
Inflammation	0
Inflammation	0
Inflammation	0
Inflammation	0
Inflammation	0
Inflammation	0
Inflammation	0
Inflammation	0
Inflammation	0
Inflammation	0
Inflammation	0
Inflammation	0
Inflammation	0
Inflammation	0
Inflammation	0
Inflammation	0
Inflammation	0
Inflammation	0
Inflammation	0
Inflammation	0
Inflammation	0
Inflammation	0
Inflammation	0
Inflammation	0
Inflammation	0

Inflammation	0
Inflammation	0
Inflammation	0
Inflammation	0
Inflammation	0
Inflammation	0
Inflammation	0
Inflammation	0
Inflammation	0
Inflammation	0
Inflammation	0
Inflammation	0
Inflammation	0
Inflammation	0
Inflammation	0
Inflammation	0
Inflammation	0
Inflammation	0
Inflammation	0
Inflammation	0
Inflammation	0
Inflammation	0
Inflammation	0
Inflammation	0
Inflammation	0
Inflammation	0
Inflammation	0
Inflammation	0
Inflammation	0
Inflammation	0
Inflammation	0
Inflammation	0
Inflammation	0
Inflammation	0
Inflammation	0
Inflammation	0
Inflammation	0
Inflammation	0
Inflammation	0
Inflammation	0
Inflammation	0
Inflammation	0
Inflammation	0
Inflammation	0

Inflammation	0
Inflammation	0
Inflammation	0
Inflammation	0
Inflammation	0
Inflammation	0
Inflammation	0
Inflammation	0
Inflammation	0
Inflammation	0
Inflammation	0
Inflammation	0
Inflammation	0
Inflammation	0
Inflammation	0
Inflammation	0
Inflammation	0
Inflammation	0
Inflammation	0
Inflammation	0
Inflammation	0
Inflammation	0
Inflammation	0
Inflammation	0
Inflammation	0
Inflammation	0
Inflammation	0
Inflammation	0
Inflammation	0
Inflammation	0
Inflammation	0
Inflammation	0
Inflammation	0
Inflammation	0
Inflammation	0
Inflammation	0
Inflammation	0
Inflammation	0
Inflammation	0
Inflammation	0
Inflammation	0
Inflammation	0
Inflammation	0
Inflammation	0

Inflammation	0
Inflammation	0
Inflammation	0
Inflammation	0
Atrophy	1
Atrophy	1
Inflammation	1
Inflammation	1
Inflammation	1
Inflammation	1
Atrophy	1
Inflammation	1
Inflammation	1
Inflammation	1
Inflammation	1
Inflammation	1
Atrophy	1
Atrophy	1
Atrophy	1
Inflammation	1
Inflammation	1
Inflammation	1
Inflammation	1
Atrophy	1
Atrophy	1
Inflammation	1
Inflammation	1
Inflammation	1
Inflammation	1
Atrophy	1
Inflammation	1
Inflammation	1
Atrophy	1
Inflammation	1
Atrophy	1
Inflammation	1
Inflammation	1
Atrophy	1
Inflammation	1
Atrophy	1
Inflammation	1
Inflammation	1
Inflammation	1
Inflammation	1

Atrophy	1
Inflammation	1
Atrophy	1
Atrophy	1
Inflammation	1
Atrophy	1
Inflammation	1
Inflammation	1
Atrophy	1
Inflammation	1
Inflammation	1
Atrophy	1
Inflammation	1
Inflammation	1
Inflammation	1
Atrophy	1
Atrophy	1
Atrophy	1
Atrophy	1
Inflammation	1
Inflammation	1
Inflammation	1
Inflammation	1
Atrophy	1
Atrophy	1
Inflammation	1
Atrophy	1
Inflammation	1
Atrophy	1
Atrophy	1
Inflammation	1
Atrophy	1
Atrophy	1
Atrophy	1
Atrophy	1
Atrophy	1
Atrophy	1
Atrophy	1
Atrophy	1
Inflammation	1
Atrophy	1
Atrophy	2
IM	2
Inflammation	2

Atrophy	2
Inflammation	2
Inflammation	2
Inflammation	2
Inflammation	2
Atrophy	2
Inflammation	2
Inflammation	2
Inflammation	2
Dysplasia	2
Inflammation	2
Inflammation	2
Inflammation	2
Dysplasia	2
Atrophy	2
Inflammation	2
Atrophy	2
Inflammation	2
Atrophy	2
Atrophy	2
IM	2
Inflammation	2
IM	2
Inflammation	2
Inflammation	2
Inflammation	2
Atrophy	2
Atrophy	2
Atrophy	2
Dysplasia	2
Inflammation	2
IM	2
Inflammation	2
Atrophy	2
Atrophy	2
Inflammation	2
Dysplasia	2
IM	2
Inflammation	2
Dysplasia	2
Inflammation	2
Inflammation	2
Atrophy	2
Atrophy	2

Atrophy	2
IM	2
Inflammation	2
IM	2
Atrophy	2
Inflammation	2
Inflammation	2
Atrophy	2
Inflammation	2
IM	2
Inflammation	2
Inflammation	2
IM	2
Inflammation	2
Inflammation	2
Inflammation	2
Atrophy	2
IM	2
Inflammation	2
Atrophy	2
Atrophy	2
IM	2
IM	2
IM	2
Inflammation	2
Atrophy	2
Inflammation	2
Inflammation	2
Atrophy	2
Inflammation	2
Inflammation	2
Atrophy	2
Inflammation	2
Inflammation	2
Inflammation	2
Inflammation	2
IM	2
Inflammation	2
Inflammation	2
Atrophy	2
Atrophy	2
Inflammation	2
Inflammation	2
Atrophy	2

Atrophy	2
Inflammation	2
IM	2
Inflammation	2
Inflammation	2
Atrophy	2
Atrophy	2
Inflammation	2
Atrophy	2
Dysplasia	2
Inflammation	2
Inflammation	2
Inflammation	2
Inflammation	2
Inflammation	2
Inflammation	2
Inflammation	2
IM	2
Atrophy	2
IM	2
Atrophy	2
Inflammation	2
Atrophy	2
Inflammation	2
Inflammation	2
Inflammation	2
Atrophy	2
Inflammation	2
Atrophy	2
Inflammation	2
IM	2
Atrophy	2
Atrophy	2
Atrophy	2
Atrophy	2
Atrophy	3
Atrophy	3
IM	3
Inflammation	3
Atrophy	3
Inflammation	3
Inflammation	3
IM	3
Atrophy	3

Inflammation	3
Atrophy	3
Atrophy	3
Inflammation	3
Atrophy	3
Atrophy	3
Atrophy	3
Atrophy	3
Inflammation	3
Atrophy	3
Atrophy	3
Atrophy	3
Inflammation	3
IM	3
Inflammation	3
Atrophy	3
Atrophy	3
Inflammation	3
Atrophy	3
Atrophy	3
Atrophy	3
IM	3
Atrophy	3
Inflammation	3
Atrophy	3
Inflammation	3
Inflammation	3
Inflammation	3
Atrophy	3
Atrophy	3
Inflammation	3
Atrophy	3
IM	3
Inflammation	3
Atrophy	3
IM	3
Inflammation	3
Atrophy	3
IM	4
Dysplasia	4
Atrophy	4
IM	4
Dysplasia	4
Atrophy	4

Dysplasia	4
IM	4
Dysplasia	4
Dysplasia	4
Dysplasia	4
Dysplasia	4
IM	4
IM	4
Dysplasia	4
Dysplasia	4
IM	4
IM	4
Dysplasia	4
IM	4
IM	4
GC	4
IM	4
